# Immune checkpoint inhibitor–associated scleroderma refractory to corticosteroids: Clinical improvement with intravenous immune globulin and dupilumab

**DOI:** 10.1016/j.jdcr.2025.12.027

**Published:** 2026-01-15

**Authors:** Dema G. Abul-Enin, Nicole L. Edmonds, Corina A. Rusu

**Affiliations:** Department of Dermatology, University of Virginia, Charlottesville, Virginia

**Keywords:** autoimmune disease, ICI-induced scleroderma, immune checkpoint inhibitors, immune-related adverse event, intravenous immune globulin, IVIG, morphea, scleroderma, steroid refractory

## Introduction

Scleroderma is an autoimmune disease characterized by increased production and deposition of collagen in the skin, which manifests as thickening and hardening of the skin. While the exact mechanism behind the disorder is unknown, few cases have been linked to immune-altering therapies, such as immune checkpoint inhibitors (ICIs).^1^ Here, we present the case of a woman who developed scleroderma after undergoing ICI therapy for mesothelioma.

## Case report

A 76-year-old woman with a past medical history of mesothelioma s/p surgical resection (2021), 2 cycles of neoadjuvant chemotherapy (2021), and immunotherapy (ipilimumab/nivolumab 2024) presented to the dermatology clinic for evaluation of a new rash in February 2025. She noted that in the past 6 months, she had developed skin thickening and discoloration with associated pruritus and burning, which was prohibiting her from continuing her immunotherapy. She had been treated with 2 rounds of low-dose oral prednisone with no improvement in her rash.

**Fig 1:**
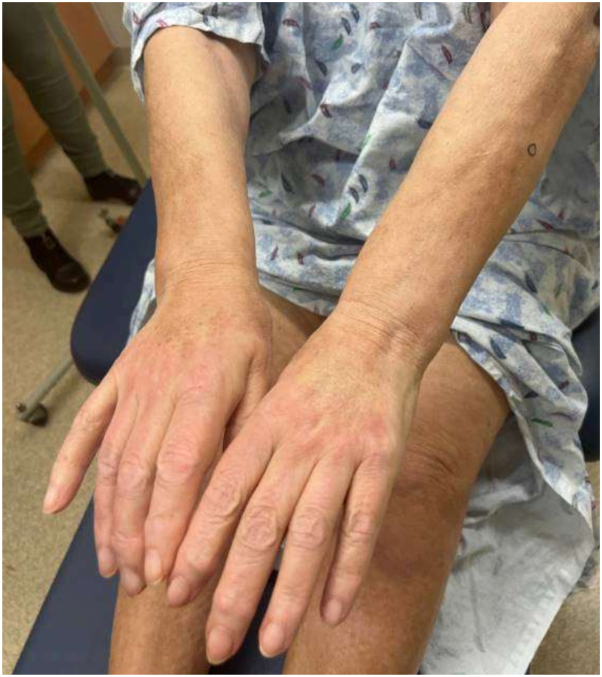
Thickened skin on patient’s hands.

**Fig 2:**
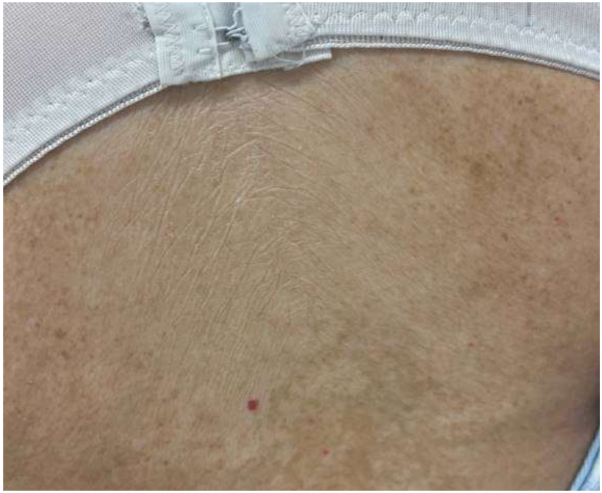
Thickened skin on patient’s midback.

**Fig 3:**
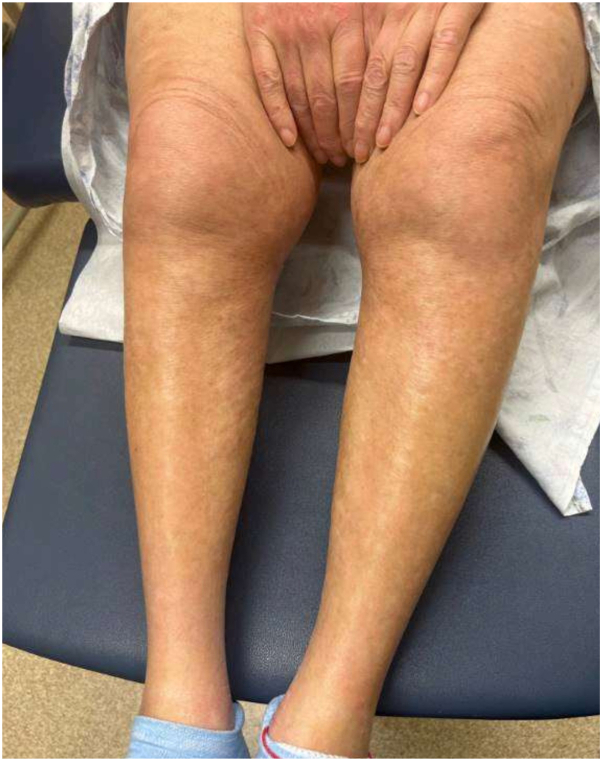
Thickened skin on patient’s legs.

**Fig 4:**
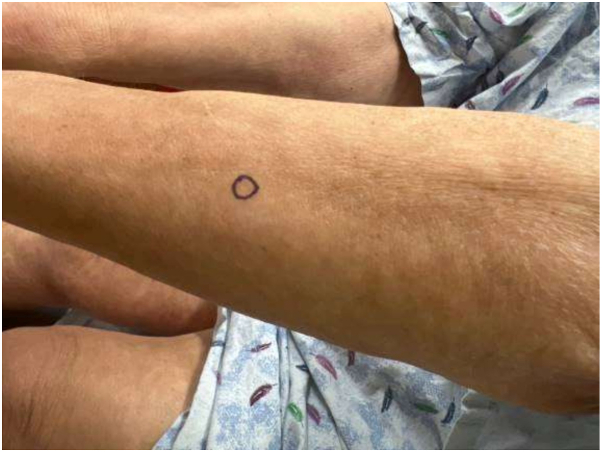
Thickened skin on patient’s left forearm, with region for punch *biopsy circled* in *black*.

**Fig 5:**
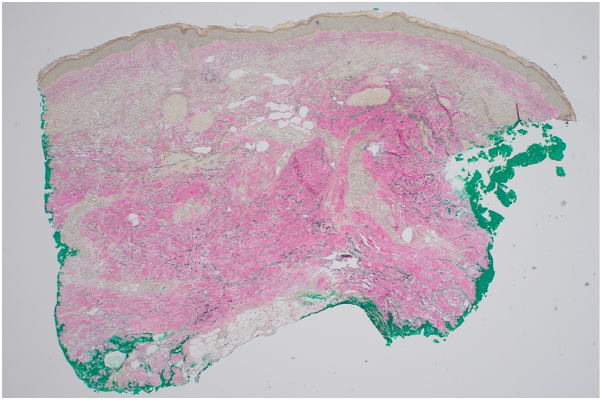
4× magnification of hematoxylin and eosin (H&E) stain on biopsy.

**Fig 6:**
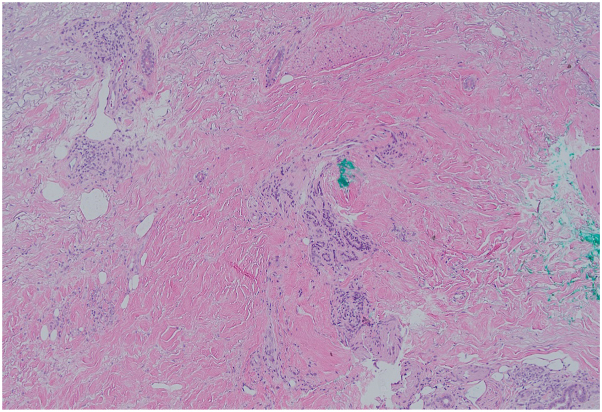
10× magnification of H&E stain on biopsy. *H&E*, Hematoxylin and eosin.

**Fig 7:**
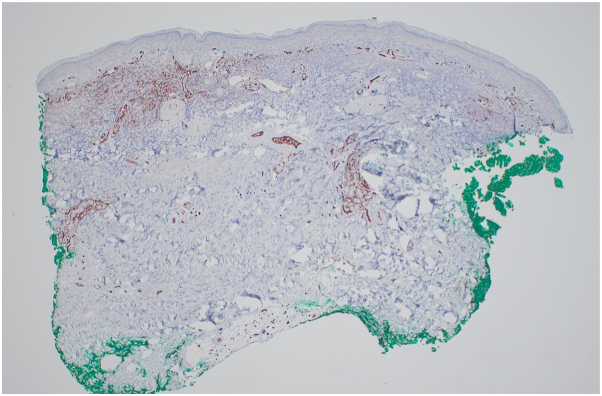
IHC staining for CD34 showing decreased expression in areas of dermal sclerosis.

**Fig 8:**
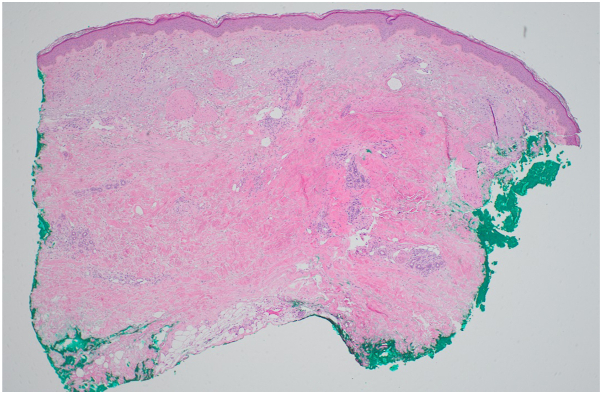
IHC staining for VVG showing disruption in elastic fibers. *VVG*, Verhoeff-Van Gieson.

On physical exam, there was tightening and hyperpigmentation of the skin on her bilateral arms and shoulders, as well as her back (Figs 1, 2 and 3). Punch biopsy taken from her left forearm demonstrates an unremarkable epidermis with underlying dermal sclerosis and loss of periadnexal adipose tissue (Figs 4, 5 and 6). There was minimal chronic inflammation, and collagen bundles appeared thickened and homogenized, extending into the deep dermis. Immunohistochemical staining for CD34 shows a marked decrease in expression within areas of dermal sclerosis, while Verhoeff-Van Gieson staining reveals disruption and fragmentation of elastic fibers (Figs 7 and 8). These histologic findings are compatible with morphea (localized scleroderma) or scleroderma.

Symptomatic treatment was started using a high-dose prednisone taper (starting at 1 mg/kg/d), and intravenous immune globulin (IVIG) was recommended. Two weeks later, prior to beginning IVIG, the patient presented again with severe pruritus that prevented sleep and a slow-healing wound around her tailbone, which the patient had treated with triamcinolone with no improvement. On physical exam, the patient had a subcutaneous hard nodule on her left lateral buttock, well-healing open wounds on the gluteal cleft and bilateral buttocks, and tightening and hyperpigmentation of the surrounding skin, consistent with progressive scleroderma. The patient was advised to continue her prednisone taper and start treatment with IVIG (6 monthly doses, 30 g per dose).

Following 8 IVIG infusions, leg and joint symptoms improved. However, the patient reported worsening of the scleroderma in her hands, with marked skin tightening that limited the use of her hands and fingers. Dupilumab (300 mg, 2 mL) was then added to combat pruritus associated with eosinophilia. This led to subsequent improvement in both pruritus and joint pain. Treatment was continued.

## Discussion

Given the close timeline between initiation of ipilimumab and nivolumab and the onset of her scleroderma as well as her biopsy results, the patient’s diagnosis was most consistent with scleroderma secondary to immunotherapy. The development of scleroderma is a rare side effect of ICI use. According to data from EudraVigilance, the European pharmacovigilance database, approximately 70 cases of scleroderma associated with ICIs have been reported. These were primarily linked to programmed cell death protein 1 (PD-1) inhibitors such as pembrolizumab and nivolumab. Notably, these cases were classified as serious, though most patients experienced favorable outcomes with appropriate management.^2^ Additionally, a 2023 literature review identified 19 cases of ICI-induced scleroderma associated with anti-PD-1 agents, 6 of which involved the use of nivolumab.^1^

Although the mechanism of action underlying ICI-induced scleroderma remains unclear, current evidence suggests that checkpoint blockade, particularly inhibition of the PD-1 pathway, disrupts normal immune tolerance. This change allows for the expansion of autoreactive effector T cells, the imbalance of regulatory T cells, and the activation of B cells.^2^, ^3^, ^4^ The resulting inflammatory cytokine environment promotes fibroblast activation and triggers the profibrotic signaling pathways that drive dermal sclerosis.^2^^,^^4^ Endothelial injury and perivascular inflammation contribute to loss of dermal CD34-positive fibrocytes and disruption of elastic fibers, findings that are consistent with our patient’s biopsy. These findings further support a pathogenic role of PD-1 pathway disruption in the development of scleroderma-like reactions.^3^^,^^5^

This mechanism may also explain previously observed alterations in PD-1 expression among patients with systemic sclerosis. In a study comparing 91 patients with systemic scleroderma and age-matched, sex-control healthy individuals, T cells and B cells in patients diagnosed with systemic scleroderma showed significantly increased PD-1 expression when compared to healthy controls. Similarly, serum sPD-1 was also found to be increased in patients with systemic scleroderma.^6^ This suggests that the introduction of agents that disturb the PD-1 and sPD-1 pathways may lead to the development of scleroderma-like symptoms, as seen in the present case.

In the literature, 2 cases have demonstrated the successful use of IVIG for ICI-induced scleroderma refractory to steroid therapy. In 1 case, a 69-year-old woman developed scleroderma-like syndrome after receiving nivolumab for metastatic small-cell lung cancer. Her symptoms improved following 10 months of IVIG combined with narrow-band UV-B therapy, and were subsequently managed with IVIG alone for 15 months.^7^ Similarly, a 66-year-old man treated with pembrolizumab for stage IV metastatic melanoma developed scleroderma, which responded to IVIG and mycophenolate mofetil (1000 mg twice daily). He reported subjective improvement in his symptoms following treatment.^8^ These cases highlight IVIG as a potential therapeutic option for managing refractory ICI-induced scleroderma when standard immunosuppressive approaches fail. Given the relative novelty of IVIG treatment for scleroderma that arises in this case, there are no clear guidelines on how to proceed with chemotherapy. In both of the aforementioned cases, the offending agent was halted while IVIG was administered.^7^^,^^8^ Moreover, according to the American Society of Clinical Oncology’s 2021 clinical practice guideline update, most Grade ≥3 immune-related adverse events require ICI interruption, with ICIs held for Grade 3 toxicities and permanently discontinued for most Grade 4 events. Additionally, American Society of Clinical Oncology notes that immunosuppressive agents beyond steroids (eg, TNF-alpha inhibitors, mycophenolate) may be required for severe cases. This indicates that ICIs should not be continued while a patient is receiving IVIG for an immune-related adverse event (typically Grade 3-4).^9^

ICI-induced scleroderma is a rare but increasingly recognized immune-related adverse event. Given the widespread use of ICIs in oncology, the prevalence may be underestimated due to underreporting, misdiagnosis, or delayed onset of symptoms. This case adds to the growing body of evidence reinforcing the need for increased clinical awareness in recognizing, managing, and treating ICI-associated autoimmune manifestations.

## Conflicts of interest

None disclosed.

## References

[1] Farrugia S., Mercieca L., Betts A., Refalo N., Boffa M.J. (2023). Scleroderma secondary to pembrolizumab: a case report and review of 19 cases of Anti-PD-1-Induced scleroderma. Case Rep Oncol.

[2] Haanen J., Ernstoff M.S., Wang Y. (2020). Autoimmune diseases and immune-checkpoint inhibitors for cancer therapy: review of the literature and personalized risk-based prevention strategy. Ann Oncol.

[3] Macklin M., Yadav S., Jan R., Reid P. (2023). Checkpoint inhibitor–associated scleroderma and scleroderma mimics. Pharmaceuticals (Basel).

[4] Yin Q., Wu L., Han L. (2023). Immune-related adverse events of immune checkpoint inhibitors: a review. Front Immunol.

[5] Nicoletti M.M., Crisci E., Cosenza V. (2024). Immune checkpoint inhibitors and scleroderma: data from the European Pharmacovigilance Database. Drugs Real World Outcomes.

[6] Fukasawa T., Yoshizaki A., Ebata S. (2017). Contribution of soluble forms of programmed death 1 and programmed death ligand 2 to disease severity and progression in systemic sclerosis. Arthritis Rheumatol.

[7] DeMaio A., Hashemi K.B., Avery A., Metcalf J.S., Winterfield L.S. (2023). A case of nivolumab-induced scleroderma-like syndrome successfully treated with intravenous immunoglobulin. JAAD Case Rep.

[8] Barbosa N.S., Wetter D.A., Wieland C.N., Shenoy N.K., Markovic S.N., Thanarajasingam U. (2017). Scleroderma induced by pembrolizumab: a case series. Mayo Clinic Proc.

[9] Schneider B.J., Naidoo J., Santomasso B.D. (2021). Management of immune-related adverse events in patients treated with immune checkpoint inhibitor therapy: ASCO guideline update. JCO.

